# Shensu IV maintains the integrity of the glomerular filtration barrier and exerts renal protective effects by regulating endogenous hydrogen sulfide levels

**DOI:** 10.3389/fphar.2024.1447249

**Published:** 2024-12-09

**Authors:** Shuhui Zhou, Liping Zheng, Tingxuan Zheng, Haiyan Zhan, Qiuyuan Lin, Jiaoao Wei, Yong Huang

**Affiliations:** ^1^ Department of Nephrology, Jiangxi University of Traditional Chinese Medicine, Nanchang, China; ^2^ Department of Nephrology, Affiliated Hospital of Jiangxi University of Traditional Chinese Medicine, Medicine Formula-Pattern Research Center of Jiangxi University of Traditional Chinese Medicine, Nanchang, China

**Keywords:** kidney disease, Shensu IV, oxidative stress, H2S, CD2AP, nephrin

## Abstract

**Background:**

Nephrotic syndrome has a significant impact on global health, often leading to cardiovascular disease and high mortality due to limited effective treatments. This study investigates the efficacy of Shensu IV in a puromycin aminonucleoside (PAN)-induced rat model of nephropathy.

**Methods:**

Rat models and *in vitro* podocyte PAN nephropathy models were established with PAN and treated with Shensu IV. Renal function was evaluated by measuring urine output and protein content, while hydrogen sulfide (H2S) and oxidative stress markers were quantified in serum and podocyte lysates. We conducted histological examination on kidney tissues and analyzed molecular markers (CD2AP, nephrin, and PI3K/AKT pathway) using RT-qPCR and Western blot.

**Results:**

Shensu IV significantly improved urine output and proteinuria, and attenuated glomerular damage, fibrosis, and mitochondrial swelling in PAN-treated rats. Mechanistically, Shensu IV enhanced endogenous H2S production, reducing oxidative stress and activating the PI3K/AKT pathway *in vivo* and *in vitro*. This facilitated the upregulation of the target genes CD2AP and nephrin, which are critical for maintaining glomerular integrity and improving renal function in PAN nephropathy models.

**Conclusion:**

Shensu IV and NaHS confer renal protection primarily by modulating oxidative stress and restoring the integrity of the glomerular filtration barrier through mechanisms involving the enhancement of the PI3K/AKT pathway and modulation of H2S levels. These findings suggest a promising therapeutic potential for these metabolites in the treatment of nephrotic syndrome.

## Graphical Abstract

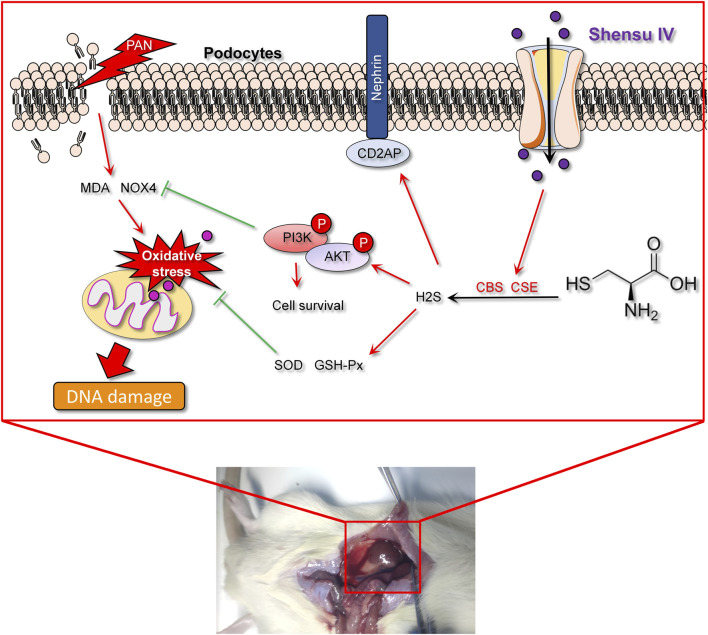

## 1 Introduction

Kidney disease is a major global public health problem, affecting millions of people and severely compromising their quality of life (Liu et al., 2019; Messerer et al., 2021). Nephrotic syndrome, a critical subtype of kidney disease, manifests clinically with severe proteinuria, edema, hyperlipidemia, and hypertension (Li G. et al., 2021). These symptoms not only escalate the risk of cardiovascular disease, but also significantly increase mortality, especially in the absence of effective treatments and in the face of progressive renal failure (Kitsou et al., 2022). Current therapeutic limitations create an urgent need for new strategies that effectively address kidney disease pathophysiology and improve patient outcomes.

**FIGURE 1:**
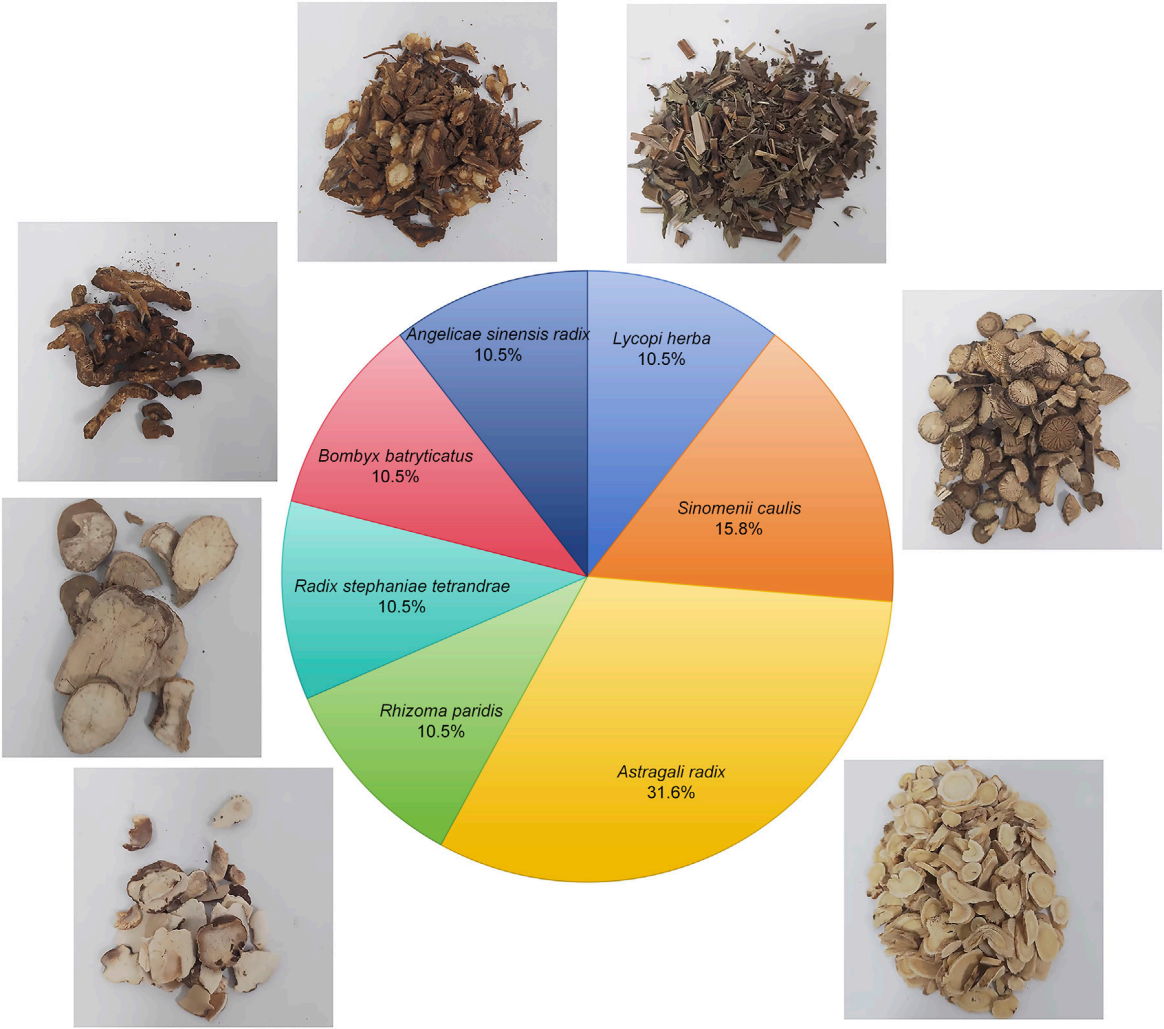
Composition of Shensu IV and relative percentage of each herbal component.

Currently, the main clinical strategies for the treatment of kidney disease include blood pressure control, reduction of proteinuria, and the use of immunosuppressive drugs (Busuioc et al., 2023). However, these treatments are often associated with side effects and are limited in their ability to prevent further deterioration of kidney function. As a result, there has been a shift in recent years toward the exploration of traditional Chinese medicine, which is known for its low side effects and multiple therapeutic targets (Lu et al., 2021; Wang H. et al., 2023). For example, Shen and colleagues have demonstrated the efficacy of traditional Chinese medicine metabolites in improving clinical symptoms, reducing proteinuria, protecting renal function, and slowing the progression of diabetic kidney disease (DKD) (Shen et al., 2024). Our previous research has confirmed that Shensu IV provides significant protection against acute kidney injury (Huang et al., 2021). Shensu IV is composed of *Astragali radix*, *Angelicae sinensis radix*, *Lycopi herba*, *Bombyx batryticatus*, *Sinomenii caulis*, *Rhizoma paridis*, and *Radix stephaniae tetrandrae* (Figure 1). Extensive research has already confirmed the potential of *Astragali radix* and *Angelicae sinensis radix* in the treatment of renal diseases (Shi et al., 2024; Wang et al., 2024), while *Lycopi herba* and *Bombyx batryticatus* are known for their significant nephroprotective effects (Han et al., 2023; Huo et al., 2020). The main active metabolite of *Sinomenii caulis*, *Sinomenine*, has been shown to attenuate renal oxidative stress, inflammation and apoptosis, thereby alleviating renal damage and fibrosis, and reducing urea and creatinine levels (Gu et al., 2023; Potocnjak et al., 2023). *Rhizoma paridis saponins*, as the major active metabolites of *Rhizoma paridis*, have been shown to possess potent anti-tumor, anti-inflammatory, and antioxidant properties (Li et al., 2023; Yang et al., 2024; Zhao et al., 2020), although their mechanisms in the treatment of renal diseases remain unclear. In addition, the anti-inflammatory, antifibrotic, and analgesic effects of *Radix stephaniae tetrandrae* have been well documented (Wang et al., 2017; Yu et al., 2001). However, the mechanisms by which Shensu IV acts in kidney disease are still poorly understood.

During the progression of kidney disease, oxidative stress, inflammation, and glomerular filtration barrier dysfunction are common pathological features (Barutta et al., 2022). Hydrogen sulfide (H2S), an endogenous gas produced by enzymes such as cystathionine beta-synthase (CBS) and cystathionine gamma-lyase (CSE), has recently been recognized for its significant role in various physiological and pathological processes (Cirino et al., 2023; Shaposhnikov et al., 2022). In renal diseases, H2S exhibits remarkable antioxidant and anti-inflammatory effects and effectively inhibits NOX4-induced oxidative stress (Han et al., 2024). As a major source of reactive oxygen species (ROS), overactivation of NOX4 is closely associated with tissue damage and fibrosis in kidney disease (Li J. et al., 2021). H2S enhances the body’s antioxidant defense systems, such as superoxide dismutase (SOD) and glutathione peroxidase (GSH-Px), helping to neutralize excessive free radicals and alleviate cell damage and inflammatory responses caused by ROS (Li et al., 2016). In addition, CD2AP helps maintain the structure of podocyte foot processes, while nephrin is a major structural metabolite of the slit diaphragm and directly regulates the selectivity of the glomerular filtration barrier (Ji et al., 2023b). Dysfunction of CD2AP and nephrin leads to disruption of the foot processes, compromising the integrity of the glomerular filtration barrier and resulting in excessive protein loss and glomerular disease; oxidative stress and inflammation can further exacerbate this damage, creating a vicious cycle (Jia et al., 2024). Therefore, by modulating the production and function of H2S, there is potential to combat oxidative stress and restore glomerular filtration barrier function, offering new therapeutic strategies for kidney disease. Increased activity of NOX4 not only promotes oxidative stress responses, but may also accelerate the pathological process in the kidney by activating inflammatory and fibrotic pathways, such as the TGF-β1 pathway (Das et al., 2014). The anti-fibrotic effect of H2S may be realized by inhibiting these pathways, thus helping to maintain the structural and functional stability of kidney tissue. However, it remains unclear whether Shensu IV can improve kidney disease by regulating endogenous H2S.

In summary, this study aimed at investigating the beneficial effects of Shensu IV and NaHS on PAN-induced nephropathy by modulating H2S production, reducing oxidative stress, and activating the PI3K/AKT pathway. The development of these treatment methods is based on a deep understanding of the molecular mechanisms of kidney disease, particularly podocyte damage, management of oxidative stress, and the potential protective role of H2S, providing a molecular basis for future preventive strategies.

## 2 Materials and methods

### 2.1 Composition and extraction of Shensu IV

Shensu IV is formulation of multiple botanical drugs, including 30 g *Astragali radix* (*Astragalus membranaceus* (Fisch.) Bunge [Fabaceae]; batch no. 220825), 10 g *Angelicae sinensis radix* (*Angelica sinensis* (Oliv.) Diels [Apiaceae]; batch no. 221017), 10 g *Lycopi herba* (*Lycopus lucidus turcz.* [Lamiaceae]; batch no. 220823), medicinal silkworms batch no. 210913), 15 g *Sinomenii caulis* (*Sinomenium acutum* (Thunb.) Rehder and E.H.Wilson [Menispermaceae]; batch no. 220714), 10 g *Bombyx batryticatus* (stiff silkworm, the dried larva of *Bombyx mori* L. infected by *Beauveria bassiana* (Bals.-Criv.) Vuill. [Bombycidae]; batch no. 210913), 10 g *Rhizoma paridis* (*paris polyphylla sm.* [Melanthiaceae]; batch no. 220620), and 10 g *Radix stephaniae tetrandrae* (*stephania tetrandra S. moore* [Menispermaceae]; batch no. 211214). These botanical drugs were obtained from Jiangzhong Traditional Chinese Medicine Co., Ltd. (Nanchang, China). The herbs were decocted three times for 40 min each time, with a solid-to-liquid ratio of 1:10 (g/mL), and then concentrated into a 100 mL stock solution, with each milliliter corresponding to 1 g of crude drug. The solution was sealed in a sterile bottle and stored at 4°C for subsequent use. For animal experiments, the stock solution was diluted 30-fold with saline and administered orally to rats.

### 2.2 Ultra-high performance liquid chromatography–mass spectrometry (UHPLC-MS) analysis

The derived solution was analyzed on using an Agilent 1290 ultra-high performance liquid chromatography (UHPLC) system (CA, United States) using a modified protocol from previous studies (Xiang et al., 2023; Zuo et al., 2022). A 2-μL aliquot was injected onto an ACQUITY UPLC HSS T3 column (1.8 μm, 2.1 × 100 mm). The mobile phase consisted of 0.2% formic acid in water (solvent A) and acetonitrile (solvent B) at a flow rate of 0.2 mL/min and a column temperature of 45°C. The gradient elution program was as follows: 0–5 min, 5%–30% B; 5–10 min, 30%–50% B; 10–15 min, 50%–70% B; 15–20 min, 70%–95% B; 20–25 min, 95%–5% B; and 25–30 min, 5% B for re-equilibration. Mass spectrometric detection was performed using an Agilent 6545 quadrupole time-of-flight (Q-TOF) mass spectrometer. Electrospray ionization (ESI) was used in both positive and negative ionization modes under the following conditions: nebulizer gas pressure of 4.0 bar, dry gas flow rate of 8 L/min, dry gas temperature of 320°C, ion accumulation time of 0.15 s, and time-of-flight of 0.6 m. The capillary voltage was set at 4.0 kV for positive ion mode and 3.5 kV for negative ion mode. Full scan mass spectrometry data were collected over a mass-to-charge (m/z) range of 100–3,000 atomic mass units (amu). Both MS/MS enhancement and MS/MS isolation modes were used to enhance the detection and identification of analytes.

### 2.3 Animals

Eighteen male Sprague-Dawley rats of SPF grade, weighing 140–160 g, were purchased from Sibeifu Biotechnology Co., Ltd. (Beijing, China), license number: SCXK (Jing) 2019-0,010. After 1 week of acclimatization, the rats were randomly divided into six groups: control, model, low-dose Shensu IV, medium-dose Shensu IV, high-dose Shensu IV, and NaHS, with three rats per group. All groups except the control were subjected to a single intravenous injection of puromycin aminonucleoside (PAN; APExBIO, Shanghai, China) to establish a model of nephropathy. Under isoflurane anesthesia, a 2-cm incision was made along the direction of the jugular vein to expose and inject 10 mg/100 g of PAN solution, followed by suturing of the incision. Postoperatively, each rat received intraperitoneal injections of potassium penicillin (1 × 10^5^ U; MeilunBio, Shanghai, China) daily for 3 days. Subsequently, the control and model groups received oral saline for 6 weeks, while the Shensu IV groups received daily oral doses of low (150 mg/kg), medium (300 mg/kg), and high (600 mg/kg) concentrations of Shensu IV, and the NaHS group received daily intraperitoneal injections of NaHS (3.136 mg/kg; Sigma-Aldrich). On the day of final intervention (day 42), all the rats survived, the experiment was terminated by euthanasia with an overdose of pentobarbital sodium (200 mg/kg), and kidney tissues were collected for pathological examination. All animal experiments were approved by the Animal Care and Use Committee of Jiangxi University of Chinese Medicine (approval number IACUC FJABR2022071101).

### 2.4 Assessment of animal metrics

Metabolic cages were used to collect and quantify urine volume from each group on days 14, 28, and 42 after the intervention. Urine protein content was measured using a protein quantification kit (C035-2-1; Jiancheng Bioengineering Institute, Nanjing, China). Blood samples or podocyte supernatants were collected on the same days to determine the levels of malondialdehyde (MDA), SOD, and H2S using their respective assay kits (MDA: A003-1, SOD: G0101W, H2S: G0133W; all from Gris Biotechnology, Suzhou, China). Optical density (OD) was measured using an enzyme-linked immunosorbent assay reader (K3; Danli Technology, Guangdong, China).

### 2.5 Pathological examination

Kidney tissues were embedded and sectioned at 4 μm using a rotary microtome. For hematoxylin and eosin (H&E) staining, the sections were immersed in hematoxylin solution (Beyotime, Shanghai, China) for 5 min and blueed in PBS (Biosharp, Anhui, China) for another 5 min. After staining with eosin (Beyotime) for 1 min, sections were dehydrated rapidly in absolute ethanol and cleared in xylene for 10 min before mounting with neutral balsam (MeilunBio). For periodic acid-Schiff (PAS) staining, sections were stained with Alcian blue (Solarbio, Beijing, China) for 10 min, washed with distilled water, then stained with Schiff reagent (Solarbio) for 20 min at room temperature in the dark, followed by rinsing with tap water for 10 min. After counterstaining with hematoxylin for 2 min and bluing in Scott’s tap water substitute (Solarbio) for 3 min, the sections were dehydrated in absolute ethanol and cleared in xylene. For Masson’s trichrome staining, the sections were stained with Weigert’s iron hematoxylin (Solarbio) for 10 min under a light shield, then blueed with Masson’s solution for 5 min, followed by three washes in PBS. Phloxine staining was performed for 1 min, washed with molybdophosphoric acid for 1 min and incubated with aniline blue for 20 s, followed by dehydration and clearing in xylene. Finally, the sections were mounted with neutral balsam.

### 2.6 Transmission electron microscopy (TEM) analysis

Kidney samples were fixed in 2.5% glutaraldehyde (Sigma-Aldrich) and post-fixed in 1% osmium tetroxide (Sigma-Aldrich) at 4°C. After dehydration through a graded series of alcohols, samples were embedded in EPON 812 resin (Electron Microscopy Sciences, PA, United States). Ultrathin sections (70 nm) were cut with a Leica Ultracut UCT microtome (Leica Microsystems, Germany) and mounted on 300 mesh copper grids. Sections were stained with uranyl acetate and lead citrate to enhance image contrast and examined under a JEM-1400 transmission electron microscope (JEOL, Tokyo, Japan), and images were captured with a Gatan digital camera (Gatan, Inc., Pleasanton, CA, United States).

### 2.7 Cell culture and treatment

Primary rat podocytes were isolated as previously described (Huang et al., 2021). Briefly, kidney tissues from three rats were digested with 0.1% collagenase IV (Gibco, Thermo Fisher Scientific, MA, United States) for 10–15 min, and the digestion was stopped with DMEM (Gibco). Cells were collected through a 100 μm mesh filter and centrifuged at 800 *g* for 5 min at 21°C. The collected cells were resuspended in DMEM supplemented with 10% fetal bovine serum and cultured at 37°C with media changes every 48 h. When the cells reached 80% confluence, they were seeded at a density of 2 × 10^5^ cells per well in 6-well plates and then treated with PAN (20 mg/mL) for 48 h to establish an *in vitro* PAN cell model. Podocytes were pretreated with Shensu IV medicated serum (10 μg/mL), NaHS (1000μM; Aladdin, Shanghai, China), ASK1 inhibitor selonsertib (100nM; APExBIO), CBS inhibitor hypericin (100μM; MeilunBio), and CSE inhibitor DL-propargylglycine (APG; 5mM; MaoKang Biotechnology, Shanghai, China) for 24 h before PAN modeling. Shensu IV medicated serum was prepared by gavaging normal rats (n = 15) with Shensu IV (3.6 mL/200 g) for 7 consecutive days. After the last dose, rats were anesthetized and blood was collected via the abdominal aorta under sterile conditions. Blood was centrifuged at 3,000 rpm for 10 min to obtain serum, which was then heat-inactivated at 56°C for 30 min and filtered through a 0.22 μm filter (MilliporeSigma, MA, United States).

### 2.8 RT-qPCR analysis

Total RNA was extracted from kidney tissue or podocytes using TRIzol reagent (Invitrogen, Thermo Fisher Scientific), and RNA concentration and purity were assessed using a NanoDrop 2000 spectrophotometer (Thermo Fisher Scientific). cDNA was synthesized using the High Capacity cDNA Reverse Transcription Kit (Applied Biosystems, Thermo Fisher Scientific) according to the manufacturer’s instructions. RT-qPCR was performed on the StepOnePlus Real-Time PCR System (Applied Biosystems) using PowerUp SYBR Green Master Mix (Applied Biosystems). The protocol included an initial denaturation at 95°C for 60 s, followed by 40 cycles of 95°C for 20 s, 56°C for 20 s, and 72°C for 38 s. GAPDH was used as an internal reference gene to normalize expression data, and relative expression levels were calculated using the 2^−ΔΔCT^ method. Primer sequences are listed in Table 1. Each sample was assayed in triplicate.

**TABLE 1:** Primer sequence for RT-qPCR.

Gene	Sequence (5′-3′)
GAPDH	F:ACGGCAAGTTCAACGGCACAG
	R:GAAGACGCCAGTAGACTCCACGAC
AKT	F:GAGGTTGCCCACACGCTTACTG
	R:GGACACAATCTCCGCACCGTAG
NOX4	F:GCCTCCATCAAGCCAAGATT
	R:TTCCAGTCATCCAGTAGAGTGTT
PI3K	F:CCGATCCTACAGTCCTATCCAAT
	R:AAGGCACAGGTCCAGAGATT
CD2AP	F:GGTGGAAAGGTGAACTGAATGGT
	R:GGTCAGGTTTTGGAGCTGGAC
Nephrin	F:AAGGATTCACGTCCGGTGAG
	R:CAGGAGAACTTGGCTCGGTT
CBS	F:CAATACCGCAACAATGGCGT
	R:TATTTCCGGGTCTGCTCACG
CSE	F:GACAAGAGCCGGAGCAATGG
	R:CCAAGCAATTCCTCGTCGGAT

### 2.9 Western blot analysis

Proteins were extracted from kidney tissue or podocytes with RIPA lysis buffer, and concentrations were determined using the BCA protein assay kit. Samples (20 g) were separated by SDS-PAGE (MeilunBio) and transferred to PVDF membranes (MilliporeSigma). Membranes were blocked with 5% skim milk (Solarbio) to prevent nonspecific binding and then incubated with primary antibodies against CD2AP (1:2000; A01756-2, BOSTER, Wuhan, China), nephrin (1:2000; A01756-2, BOSTER), CBS (1:10,000; 14787-1-AP, Proteintech, Wuhan, China), CSE (1: 4,000; 12217-1-AP, Proteintech), PI3K (1:2000; 60225-1-Ig, Proteintech), p-PI3K (1:2000; bs-3332R, BOSTER), AKT (1:10,000; 60203-2-Ig, Proteintech), p-AKT (1: 10,000; 66,444-1-lg, Proteintech), NOX4 (1:8,000; 14347-1-AP, Proteintech), and GAPDH (1:40,000; 60004-1-Ig, Proteintech). Bound antibodies were detected with HRP-conjugated secondary antibodies (goat anti-rabbit IgG (H + L), 1:10,000; SA00001-1, Proteintech) and visualized with the ECL detection system (MeilunBio). Protein expression was analyzed using Image Lab software (Bio-Rad, CA, United States).

### 2.10 Data analysis

All data were statistically analyzed using GraphPad Prism 8 (GraphPad Software, CA, United States). Group comparisons were performed using one-way analysis of variance (ANOVA), followed by Tukey’s test for multiple comparisons. The significance level for all statistical tests was set at *p* < 0.05.

## 3 Results

### 3.1 Shensu IV improved urine volume and urinary protein in PAN rats

**FIGURE 2:**
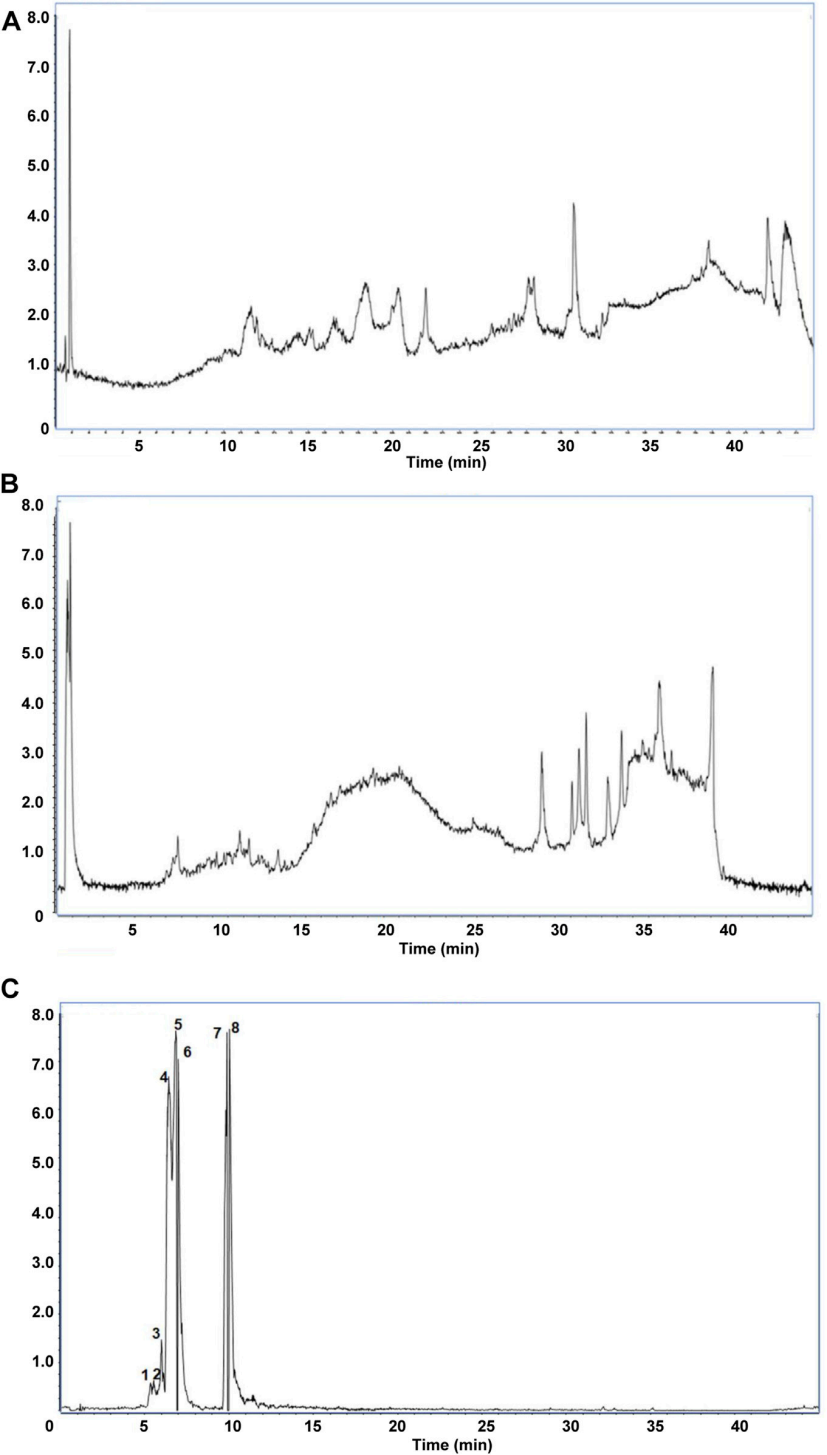
The main components in Shensu IV as detected by LC-MS. **(A)** Negative ion mode total ion chromatogram (TIC). **(B)** Positive ion mode TIC. **(C)** Base peak ion chromatography and corresponding compounds. 1: l-Carnitine; 2: l-Carnitine, P-lysoPC (LPC) 16:0; 3: Coumaroyl tyramine; 4: Tetramethylpyrazine; 5: LPC 18:1; 6: Choline; 7: (S,S)-Butane-2,3-diol; 8: Scopoletin.

**FIGURE 3:**
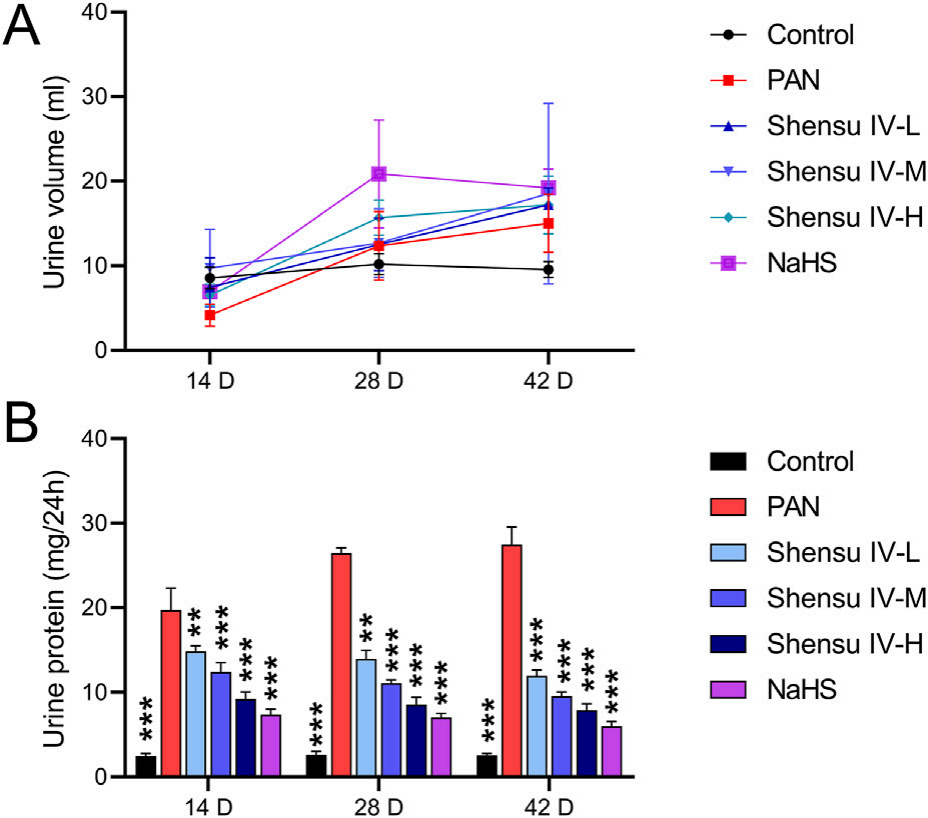
Impact of Shensu IV and NaHS on Urine Output and Protein Levels in PAN-treated Rats. **(A)** Changes in urine volume over time. Graph shows the daily urine output measured on Days 14, 28, and 48 post-treatment in control, PAN only, and PAN rats treated with Shensu IV and NaHS. **(B)** Urinary protein concentrations at Days 14, 28, and 48. Data represent urinary protein levels measured in control, PAN only, and PAN rats treated with different doses of Shensu IV and NaHS. **P< 0.01, ***P< 0.001. Abbreviations: PAN, Puromycin Aminonucleoside; NaHS, Sodium Hydrosulfide.

We first identified the active metabolites of Shensu IV (Figure 2; Table 2). After oral administration of Shensu IV in the model group, urine volume data showed that on day 14 post intervention, PAN-treated rats had significantly reduced urine output compared to normal rats. However, by days 28 and 48, the urine volume of PAN-treated rats exceeded that of controls. Notably, under the intervention of Shensu IV and NaHS from days 14–48, there was a significant increase in urine output in PAN-treated rats (*p* < 0.05) (Figure 3A). In addition, Urinary protein levels were elevated in PAN rats on days 14, 28, and 48; treatment with Shensu IV and NaHS significantly reduced these levels (*p* < 0.001) (Figure 3B).

**TABLE 2:** The retention time (RT), molecular weight, and MS data of the identified peaks.

No.	Metabolites	RT (min)	MW	MS
1	L-Carnitine	0.77695	161.2	162.1129
2	LPC 16:0	26.34937	495.63	496.3426
3	Coumaroyl tyramine	13.11965	283.32	284.1285
4	Tetramethylpyrazine	4.503284	136.19	137.111
5	LPC 18:1	27.22265	521.3	522.3564
6	Choline	0.8071333	104.17	104.1091
7	(S,S)-Butane-2,3-diol	12.07172	90.12	73.06499
8	Scopoletin	6.421317	192.17	193.0511

### 3.2 Shensu IV improves oxidative stress and H2S levels in PAN rats

**FIGURE 4:**
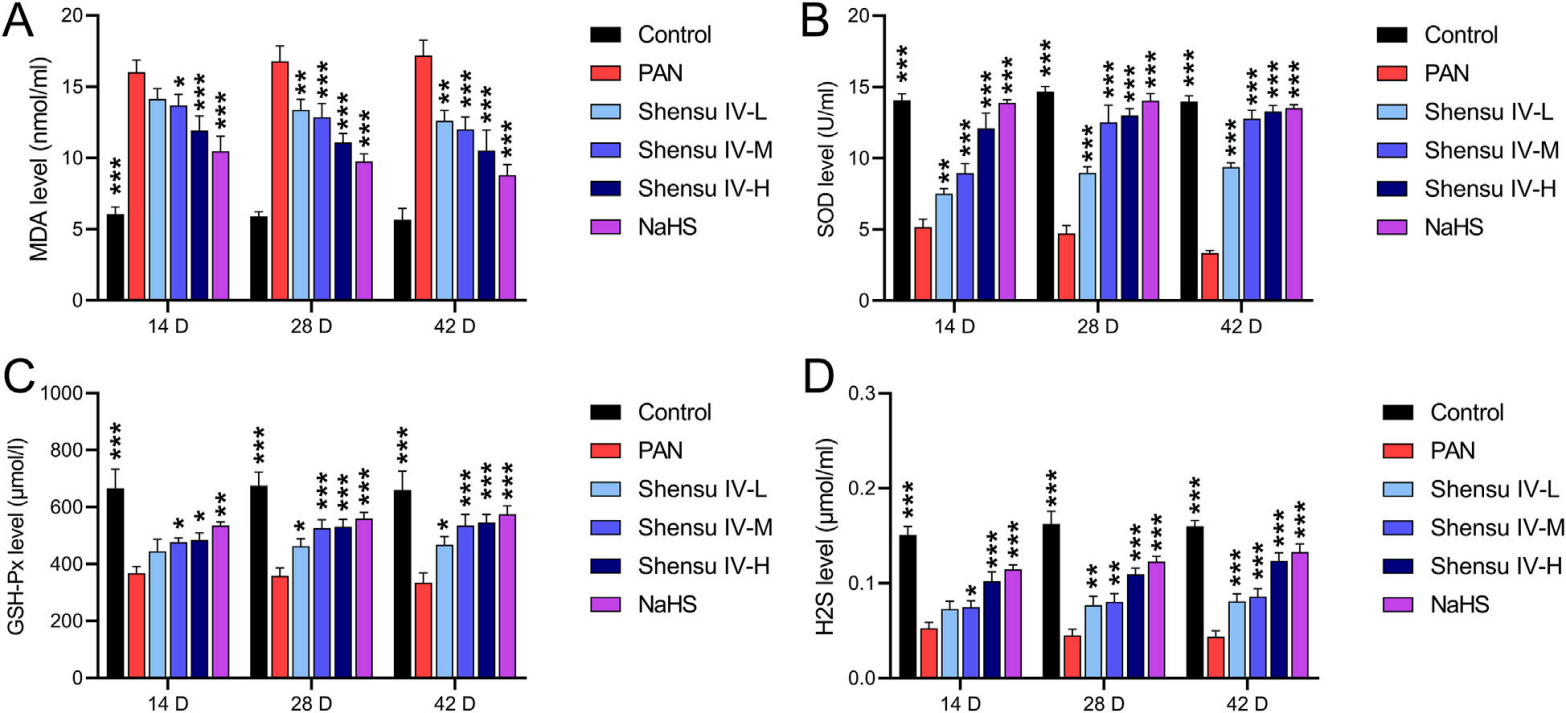
Shensu IV reduces oxidative stress and increases H2S content in PAN rats. The effects of Shensu IV on MDA **(A)**, SOD **(B)**, GSH-Px **(C)** and H2S **(D)** in serum of PAN rats were detected. *P< 0.05, **P< 0.01, ***P< 0.001. Abbreviations: MDA, Malondialdehyde; SOD, Superoxide Dismutase; GSH-Px, Glutathione Peroxidase; H2S, Hydrogen Sulfide.

On days 14, 28, and 48, serum MDA levels were significantly elevated in PAN rats, whereas Shensu IV dose-dependently reduced these levels, with NaHS showing superior efficacy (Figure 4A). In addition, compared with normal rats, PAN rats showed significantly reduced serum levels of SOD, GSH-Px and H2S, which were significantly improved by Shensu IV and NaHS treatments, especially at the highest dose of Shensu IV, which was comparable to the effects of NaHS (Figures 4B–D).

### 3.3 Shensu IV improved renal tissue pathology in PAN rats

**FIGURE 5:**
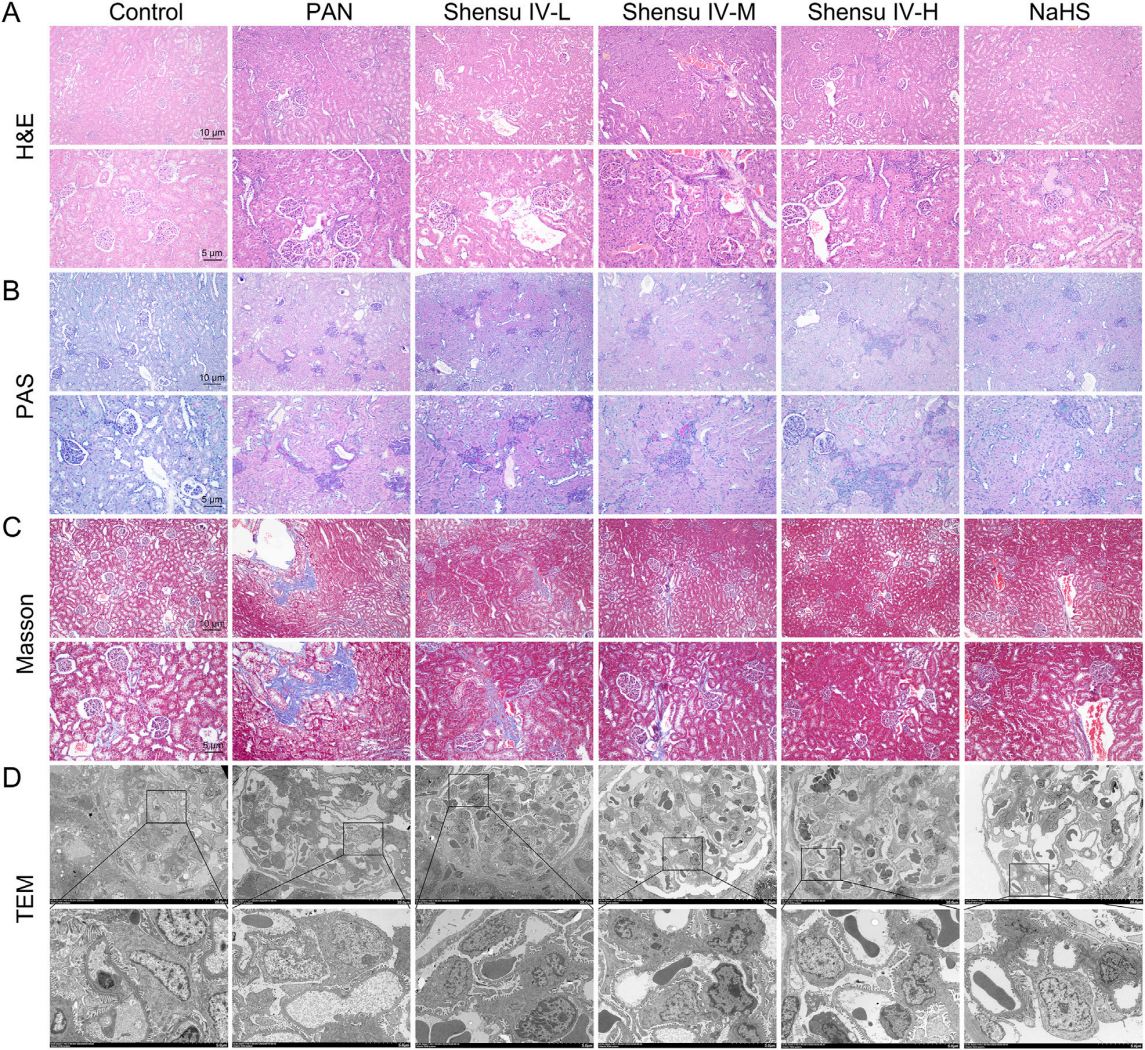
Shensu IV improved renal tissue pathology in PAN rats. **(A)** H&E staining was used to detect the effect of Shensu IV and NaHS on renal lesions in PAN rats. **(B)** PAS staining was used to detect the effect of Shensu IV and NaHS on the glycogen of PAN rats. **(C)** Masson staining was used to detect the effect of Shensu IV and NaHS on renal fibrosis in PAN rats. **(D)** The effect of Shensu IV and NaHS on mitochondrial morphology in renal tissue of PAN rats was detected by TEM. Abbreviations: H&E, Hematoxylin and Eosin; PAS, Periodic Acid-Schiff; TEM, Transmission electron microscopy.

H&E staining analysis of the effects of Shensu IV revealed that compared with controls, PAN rat kidneys exhibited lobulated glomeruli with mesangial and basement membrane thickening and partial involvement of tubular basement membrane proliferation with varying degrees of inflammatory cell infiltration. Treatment with low, medium, and high doses of Shensu IV ameliorated these histopathologic changes, and NaHS alleviated the symptoms of plasma protein leakage (Figure 5A). PAS staining showed an increased glycogen content in the kidneys of PAN rats compared with normal rats; however, this was significantly reduced by treatment with Shensu IV and NaHS, especially at the highest dose of Shensu IV, which closely matched the effects of NaHS (Figure 5B). Masson’s staining showed that PAN rats had significantly more collagen deposition and obvious fibrosis in the renal tissues compared to normal rats. This fibrosis was attenuated by treatment with all doses of Shensu IV and NaHS, especially at the highest dose of Shensu IV, where the effects closely paralleled those of NaHS (Figure 5C). TEM of the renal cortex in PAN rats showed mitochondrial swelling, partial loss of pedicles, and vacuolization in glomerular podocytes; these changes were significantly reversed by Shensu IV treatment, especially at the highest dose, which showed similar results to NaHS (Figure 5D).

### 3.4 Shensu IV regulates the PI3K/AKT signaling pathway through H2S

**FIGURE 6:**
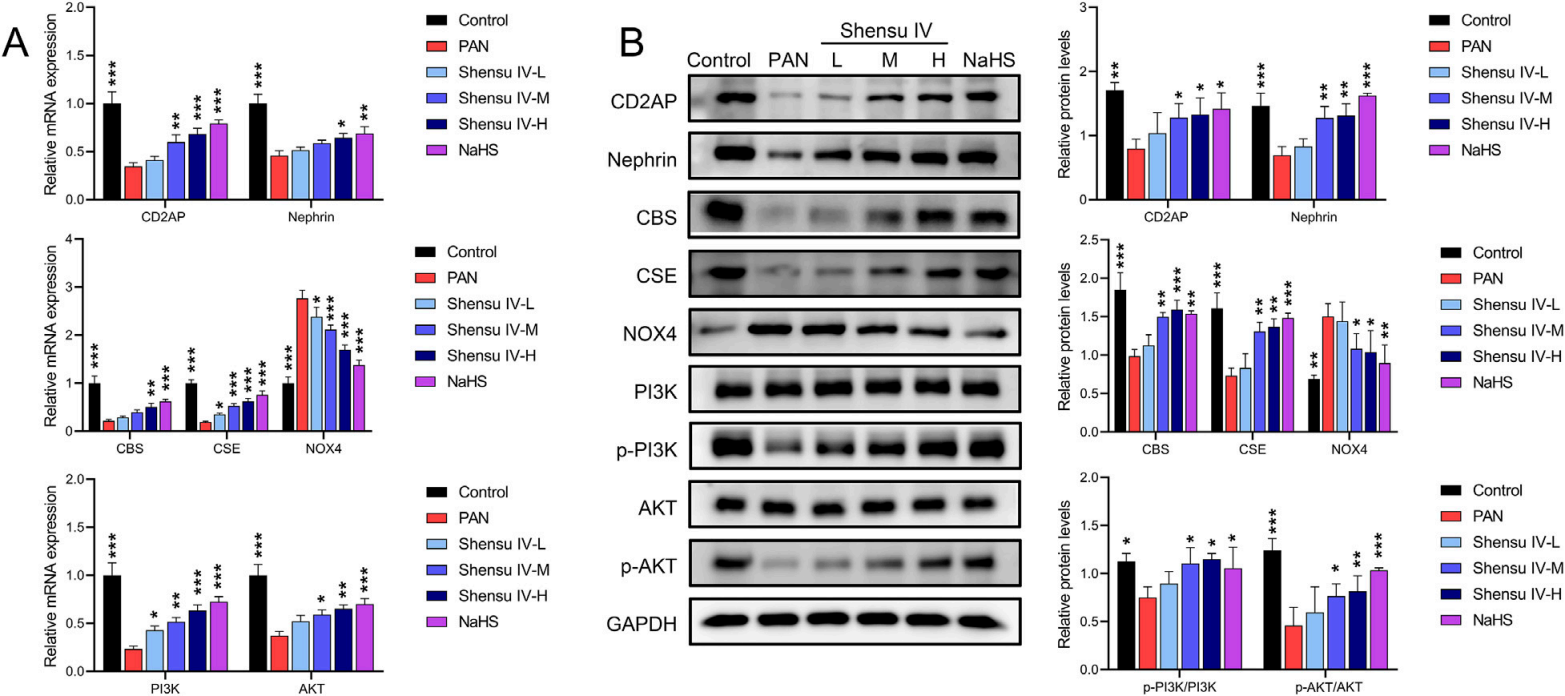
Shensu IV regulates the PI3K/AKT signaling pathway through H2S. **(A)** The effects of Shensu IV and NaHS on the mRNA expression of CD2AP, nephrin, CBS, CSE, NOX4, PI3K, and AKT in renal tissue of PAN rats were analyzed by RT-qPCR. **(B)** Western blot analysis of the effects of Shensu IV and NaHS on the protein levels of CD2AP, nephrin, CBS, CSE, NOX4, PI3K, p-PI3K,AKT,p-AKT in renal tissue of PAN rats. *P< 0.05, **P< 0.01, ***P< 0.001. Abbreviations: CD2AP, CD2-associated protein; CBS, Cystathionine β-synthase; CSE, Cystathionine γ-lyase; PI3K, Phosphoinositide 3-Kinase; AKT, Protein Kinase B.

RT-qPCR and Western blot analyses revealed that PAN rats had decreased mRNA and protein levels of CD2AP, nephrin, CBS and CSE and increased levels of NOX4 mRNA and protein compared to normal rats. Treatment with Shensu IV and NaHS significantly ameliorated these changes; the highest dose of Shensu IV showed effects similar to NaHS. In addition, key genes in the PI3K/AKT pathway in PAN rat kidneys, including PI3K and AKT, showed significant reductions in expression and phosphorylation, which were dose-dependently increased by Shensu IV treatment; the highest dose of Shensu IV showed effects similar to those of NaHS (Figure 6).

### 3.5 Shensu IV improves oxidative stress and H2S levels in podocytes

**FIGURE 7:**
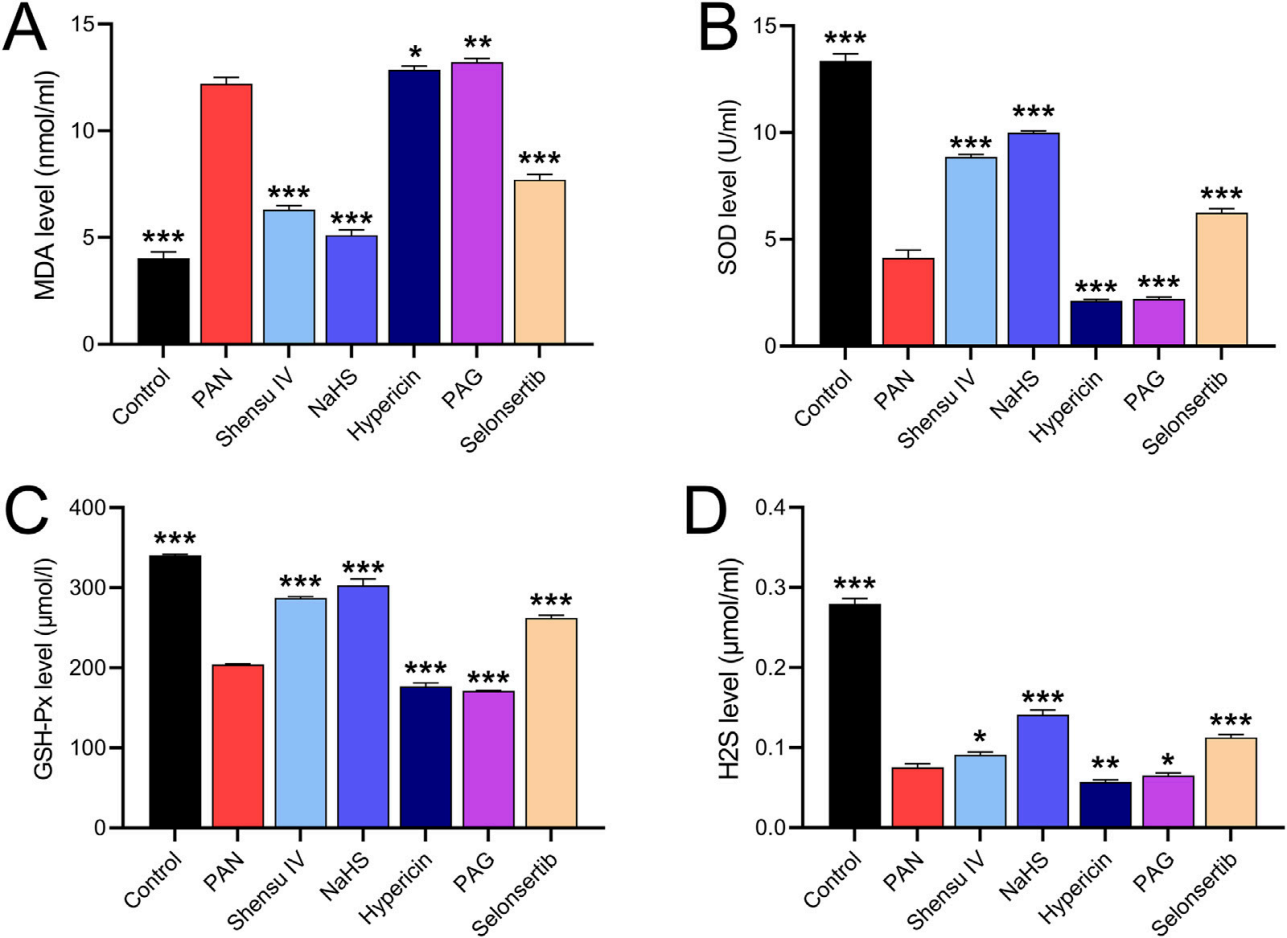
Shensu IV reduces oxidative stress and increases H2S content in podocytes. The effects of Shensu IV on MDA **(A)**, SOD **(B)**, GSH-Px **(C)** and H2S **(D)** in PAN-induced podocyocytes were detected. *P< 0.05, **P< 0.01, ***P< 0.001. Abbreviations: MDA, Malondialdehyde; SOD, Superoxide Dismutase; GSH-Px, Glutathione Peroxidase; H2S, Hydrogen Sulfide.

*In vitro* studies of the effects of Shensu IV on rat podocytes showed that PAN increased MDA and decreased SOD, GSH-XP and H2S levels compared to normal cells. These effects were attenuated by Shensu IV, NaHS and selonsertib; hypericin and APG further enhanced the effects of PAN (Figure 7).

### 3.6 Shensu IV regulates the PI3K/AKT pathway through H2S in podocytes

**FIGURE 8:**
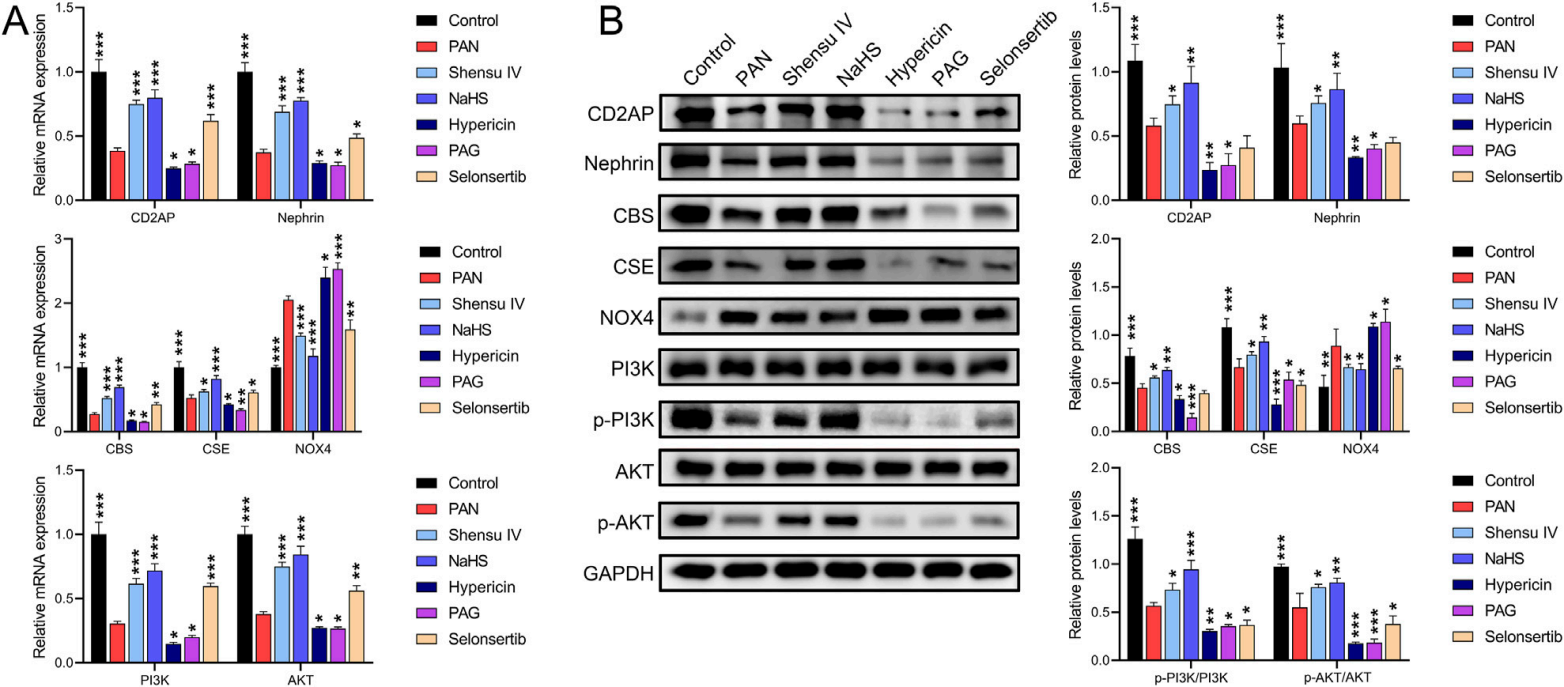
Shensu IV regulates the PI3K/AKT signaling pathway through H2S in podocytes. **(A)** The effects of Shensu IV and NaHS on the mRNA expression of CD2AP, nephrin, CBS, CSE, NOX4, PI3K, and AKT in podocytes were analyzed by RT-qPCR. **(B)** Western blot analysis of the effects of Shensu IV and NaHS on the protein levels of CD2AP, nephrin, CBS, CSE, NOX4, PI3K, p-PI3K,AKT,p-AKT in PAN-induced podocyocytes. *P< 0.05, **P< 0.01, ***P< 0.001. Abbreviations: CD2AP, CD2-associated protein; CBS, Cystathionine β-synthase; CSE, Cystathionine γ-lyase; PI3K, Phosphoinositide 3-Kinase; AKT, Protein Kinase B.

RT-qPCR and Western blot results showed that in rat podocytes, Shensu IV and NaHS significantly increased the PAN-mediated reduction of mRNA and protein levels of CD2AP, nephrin, CBS, and CSE, and also increased the mRNA levels and phosphorylation of PI3K and AKT (Figures 8A,B). In addition, Shensu IV, NaHS, and selonsertib significantly suppressed PAN-induced increases in NOX4 mRNA and protein levels; hypericin and APG further promoted the effects of PAN (Figures 8C,D). Selonsertib did not show significant intervention effects on the protein levels of CD2AP, nephrin, CBS, or the phosphorylation of PI3K/AKT.

## 4 Discussion

Kidney disease often presents without significant symptoms in its early stages, potentially leading to undetected complications. Commonly prescribed medications to manage such conditions include ACE inhibitors and angiotensin receptor blockers (ARBs), which help lower blood pressure and slow the loss of kidney function (Bhandari et al., 2022). However, long-term use of these drugs can lead to side effects that reduce patients’ quality of life and life expectancy (Bell and Jerkins, 2024). In response, this study explores the development of a new synthetic traditional Chinese medicine, Shensu IV, designed to protect kidney tissue.

The Shensu herbal formulations, including Shensu I, II, and III, have been traditionally used in certain regions of southern China for the treatment of chronic kidney disease. However, a systematic analysis of their pharmacological efficacy has not been conducted. Based on Shensu III, our team prepared Shensu IV by improving the combination and dosage of botanical drugs. This improved formulation was tested in a mouse model of PAN-induced nephropathy to investigate its therapeutic effects and underlying mechanisms. The PAN-induced nephropathy is a valuable model for studying glomerular diseases and understanding molecular mechanisms and progression of nephropathy (Lee et al., 2022; Nguyen et al., 2022). Changes in urine output serve as an early indicator of renal injury, reflecting the kidney’s ability to regulate water and electrolyte balance (Siregar et al., 2023). Our results suggest that the initial decrease in urine output induced by PAN treatment may be related to tubular damage, while interventions with Shensu IV and NaHS effectively restored this function, suggesting potential protection against the toxic effects of PAN. The significant reduction in urinary protein further supports the role of Shensu IV and NaHS in preserving glomerular integrity. Proteinuria, a direct consequence of glomerular injury, is typically associated with glomerular basement membrane injury and podocyte dysfunction (Long et al., 2022). These findings are consistent with previous studies of PAN-induced nephropathy in which glomerular injury and tubular lesions are considered the primary causes of renal failure (Shen et al., 2020; Yamanaka et al., 2023).

A key factor contributing to this damage is a sharp increase in renal oxidative stress levels (Liu et al., 2023). Evidence suggests that H2S can enhance the activities of GSH-Px and SOD, thereby helping to inhibit ROS produced by NOX4 and thus reduce oxidative stress levels as indicated by MDA (Kamat et al., 2015; Yuksel et al., 2022). Our application of exogenous H2S (NaHS) in PAN rats resulted in a significant reduction in oxidative stress levels in serum or *in vitro* podocyte models, as evidenced by decreased MDA levels and increased SOD and GSH-Px levels, with downregulation of NOX4 expression, supporting this hypothesis. Importantly, Shensu IV increased endogenous H2S, and restored the expression of CBS and CSE, thereby reducing oxidative stress. This suggest that H2S is presumably involved in the antioxidant-mediated renoproetective effect. This antioxidant effect likely helps protect glomerular structure and function by reducing oxidative stress.

Further pathological analysis revealed the protective effects of Shensu IV and NaHS on renal tissue structure. In the PAN rat model, the observed lobulated structure of glomeruli, basement membrane thickening and collagen deposition were alleviated after intervention. These changes are likely related to the regulation of oxidative stress and fibrotic processes in renal cells by Shensu IV and NaHS, as confirmed by a reduction in collagen observed by Masson’s staining (Patera et al., 2024). CD2AP contributes to the formation and maintenance of the glomerular filtration barrier by interacting with nephrin and other proteins such as podocin on the slit diaphragm of podocytes (Ji et al., 2023a). Under oxidative stress, these proteins may be abnormally expressed, compromising the structural and functional integrity of podocytes (Chen et al., 2021). TEM observations of mitochondrial swelling and disappearance of foot processes in rat renal cortical podocytes indicate significant cellular damage. An important finding of this study is that treatment with Shensu IV and NaHS resulted in some recovery of podocyte ultrastructure, especially at higher doses of Shensu IV, likely due to their antioxidant properties that help mitigate cell damage caused by oxidative stress. In addition, our findings of upregulated levels of CD2AP and nephrin in PAN rats or *in vitro* podocyte models further confirm that Shensu IV and NaHS contribute to the integrity of podocyte structures.

Previous studies have shown that the PI3K/AKT signaling pathway can promote renal recovery by activating CD2AP and nephrin (Zheng et al., 2020), while blocking this pathway significantly inhibits the synthesis of CSE and CBS (Liu et al., 2017). At the molecular level, we observed that Shensu IV and NaHS activate the PI3K/AKT pathway and significantly increase the expression of proteins such as CD2AP, nephrin, CBS, and CSE in PAN rat kidneys or *in vitro* podocytes, which is consistent with previous findings that H2S activates the PI3K/AKT pathway (Lin et al., 2020; Zhu et al., 2022). The PI3K/AKT pathway plays a critical role in maintaining cell survival, inhibiting apoptosis, and regulating cell metabolism, and changes in its activity may represent one of the key mechanisms by which Shensu IV and NaHS protect renal function (Liu et al., 2018). Our results with the CBS inhibitor hypericin and the CSE inhibitor APG in an *in vitro* PAN model show similar effects, reducing endogenous H2S production, which mediates further reductions in CD2AP and nephrin expression and promotes further oxidative stress. The potential of the ASK1 inhibitor selonsertib in the treatment of fibrotic diseases significantly increases endogenous H2S production and reduces oxidative stress, which is also supported by its effects on suppressing NOX4 expression. In addition, the effects of selonsertib on the PAN cell model after intervention promote mRNA levels of CD2AP, nephrin, CBS, CSE, PI3K, AKT, but inhibit CSE protein levels and phosphorylation of PI3K and AKT. This suggests that the inhibition of ASK1 by selonsertib may activate a negative feedback mechanism leading to regulatory phosphorylation changes of the pathway itself, or it may be a result of the redox state and cellular stress responses with its promotion of H2S and inhibition of oxidative stress not solely mediated by the PI3K/AKT pathway (Wang D. et al., 2023; Wen et al., 2021).

This study has limitations. First, there is a lack of relevant clinical data to support the renoprotective effects of Shensu IV. Second, many relevant target genes or pathways were not investigated in this study, and as a newly developed combination of traditional Chinese medicine, further research may be needed to substantiate its efficacy. Third, the PAN nephropathy model cannot fully replicate the specific conditions of *in vivo* kidney injury or other nephropathy models, such as diabetic nephropathy. Four, the use of only three rats per group in the animal experiments conducted in this study may limit the statistical analysis of the therapeutic effects of Shensu IV. Finally, we have not analyzed the potential toxicity or long-term side effects of Shensu IV, which are important factors limiting the clinical use of drugs. These areas will be the focus of our future research.

In conclusion, our study demonstrates the potential efficacy of Shensu IV in regulating oxidative stress responses, ameliorating renal tissue pathology and function, and modulating protein expression through the PI3K/AKT signaling pathway, achieved by stimulating the endogenous H2S pathway. These findings underscore the potential of Shensu IV as a promising therapeutic agent for kidney diseases characterized by oxidative stress and podocyte injury, providing a theoretical basis for its clinical application in the treatment of nephropathy.

## Data Availability

The datasets used as well as analyzed for this study will be available from the corresponding author upon reasonable request.
